# Non-invasive Prenatal Testing: Technologies, Clinical Assays and Implementation Strategies for Women’s Healthcare Practitioners

**DOI:** 10.1007/s40142-013-0010-x

**Published:** 2013-03-17

**Authors:** Amy Swanson, Amy J. Sehnert, Sucheta Bhatt

**Affiliations:** Department of Clinical Affairs, Verinata Health, an Illumina Company, 800 Saginaw Drive, Redwood City, CA 94063 USA

**Keywords:** Cell-free DNA (cfDNA), Fetal aneuploidy, Sex chromosome aneuploidies, Massively parallel sequencing, Non-invasive prenatal testing (NIPT), Prenatal testing, Next-generation sequencing, Trisomy 21, Trisomy 18, Trisomy 13, Prenatal

## Abstract

The field of prenatal genetic testing has exploded with new non-invasive technologies and test options in the past several years. It is challenging for women’s healthcare providers to keep up with the multitude of publications and provide patients with the most accurate and up-to-date information possible regarding prenatal testing. In this article, we examine the sequencing technologies that provide the framework for non-invasive prenatal testing (NIPT) and review the major North American NIPT clinical validation studies published in 2011 and 2012. This paper also compares and contrasts the commercially available non-invasive prenatal tests in the United States, discusses clinical implementation recommendations from professional societies and highlights considerations for genetic counseling.

## Introduction

In 1959, Dr. Jerome Lejeune established trisomy 21 (three copies of chromosome 21) as the cause of Down syndrome. This was the first time that the etiology of a clinical disorder was found to be caused by a chromosome abnormality. By 1960, trisomy 18 had been determined to be the cause of Edwards syndrome and trisomy 13 the cause of Patau syndrome. Since these findings, the clinical significance of numerical chromosome aberrations has been well established. In humans, approximately 10–30 % of fertilized eggs have an incorrect number of chromosomes (aneuploidy). An estimated one-third of all miscarriages are aneuploid and one in every 300 liveborns is aneuploid. As such, aneuploidy is the leading known genetic cause of miscarriage and congenital birth defects [1, 2].

Pregnant women considered to be at high risk have been offered prenatal diagnosis to detect chromosomal disorders since the late 1960s. Amniocentesis and chorionic villus sampling (CVS) are the most common methods available for diagnostic testing; both of these are invasive procedures and pose risks to the mother and fetus (most significantly a risk of miscarriage of 1 in 200–400 and 1 in 100–200, respectively) [3, 4]. More recently, one study reported a 1 in 1600 risk of miscarriage following amniocentesis [5]. While the actual procedure-related miscarriage rates are debatable, most practitioners agree that there is some inherent risk. Due to these risks, common practice is that healthcare providers recommend the option of diagnostic testing only to women at high risk of having a chromosomally abnormal fetus. Initially, determination of a high-risk population was based on maternal age alone, and hundreds of invasive procedures were performed to find one affected fetus. Selection of such women has improved over the years through a combination of measurement of maternal serum markers and most recently with the measurement of fetal nuchal translucency (NT) by ultrasound. Various screening tests to determine risk for fetal trisomy 21 are currently available including measurement of: NT only, serum-only (in the first and/or second trimester) or the combination of NT and serum markers. Using these methods, detection rates vary from 70–94 % with false positive rates of 1–5 % depending on the screen performed, the gestational age at the time it is performed and maternal age [6]. Most laboratories also provide screening results for trisomy 18 and, less often, trisomy 13. Overall, these methods are limited by their sub-optimal sensitivities and specificities and often involve a multistep testing process including an ultrasound measurement that can only be performed at centers with certified sonographers.

For decades, researchers, physicians and pregnant women alike have searched for a non-invasive way to perform prenatal diagnosis. Ideally, such a non-invasive prenatal test would replace amniocentesis and CVS or at least minimize false positive results, thus significantly reducing the number of women undergoing unnecessary, risky invasive procedures [7].

The past several years have seen many exciting advances in the field of non-invasive prenatal testing (NIPT) including the discovery of fetal cell-free DNA (cfDNA) in maternal plasma and the development of massively parallel sequencing (MPS) and counting techniques using cfDNA, leading to the launch of the first non-invasive tests for fetal aneuploidy. But, the work is not over yet. In October 2010, a cfDNA testing survey was completed by 62 women’s healthcare providers; 87 % of respondents were physicians and 11 % were nurse practitioners, registered nurses or certified nurse-midwives. A significant finding from this survey was only 15 % of participants reported having a “high level of knowledge” about NIPT [8]. In December 2012, the American College of Obstetricians and Gynecologists issued a joint committee opinion with the Society of Maternal Fetal Medicine supporting the use of NIPT in clinical practice for high-risk women [9••]. It is now the responsibility of women’s healthcare providers to become more educated about NIPT.

The aim of this paper, therefore, is to inform women’s healthcare providers about the principles of NIPT so they may determine if and when it is right for their patients and how to counsel these women both before and after undergoing NIPT.

## Properties of Fetal Cell-Free Nucleic Acids

The existence of cfDNA in blood was first discovered in 1947 [10]. This cfDNA is present in small fragments of 150–200 base pairs in length [11, 12]. These fragments are most likely degraded nuclear DNA from cells that have undergone programmed cell death (also known as cellular apoptosis) [13, 14•], although other hypotheses for the origin of cfDNA (such as spontaneous release by living cells) have also been proposed [15, 16]. Despite the elusiveness of the cfDNA origin, analyzing the cfDNA for diagnostic purposes was first motivated by the finding of tumor-derived cfDNA in cancer patients [17, 18]. The subsequent discovery in 1997 of cfDNA fragments of fetal Y-chromosomes in the plasma of pregnant women with male fetuses opened the door for the development of NIPT using maternal blood [19].

Studies summarized in the reviews by Bianchi [20] and Edlow and Bianchi [21•] suggest that the majority of fetal cfDNA in maternal plasma is derived from the placenta, with minor contributions from the fetal hematopoietic system and the fetus itself. Fetal cfDNA can be reliably detected in the maternal circulation by 7 weeks gestation and its amount increases with gestational age. The portion of fetal cfDNA is called the fetal fraction. The fetal fraction varies among pregnant individuals but has been shown to be 10 % on average (ranging from 3 to 19 %) [22, 23]. One study suggested that fetal cfDNA has an average half-life of 16.3 min (ranging from 4 to 30 min) and is cleared rapidly post-delivery such that levels are undetectable by a few hours post-partum [24]. All of these properties make fetal cfDNA an ideal candidate for NIPT.

Early clinical applications of fetal cfDNA for prenatal testing included fetal RhD genotyping [25] and sex determination to aid in the risk assessment of X-linked disorders and congenital adrenal hyperplasia (CAH) utilizing the detection of Y-chromosomes in the plasma of women pregnant with male fetuses [26]. The initial success in fetal RhD genotyping and fetal sex determination demonstrated the utility of fetal cfDNA in NIPT and encouraged the research and development of other non-invasive assays for monogenetic disorders, such as thalassemia, Huntington disease, cystic fibrosis, and myotonic dystrophy [21•]. However, most of the developed assays are PCR-based and thus are limited by PCR primer specificity and assay sensitivity. Also, given that most cfDNA in circulation is maternal in origin and there is over 99 % homology between maternal and fetal DNA, it is difficult to use the traditional PCR technology for fetal aneuploidy detection. Therefore, a novel approach for developing NIPT to detect fetal aneuploidy was still needed. The recent advancements in DNA sequencing technology, as well as counting statistics, have provided a timely opportunity to develop new methods for the non-invasive detection of fetal aneuploidy.

## Sequencing Methodologies and Bioinformatic Analytical Approaches

**Fig. 1:**
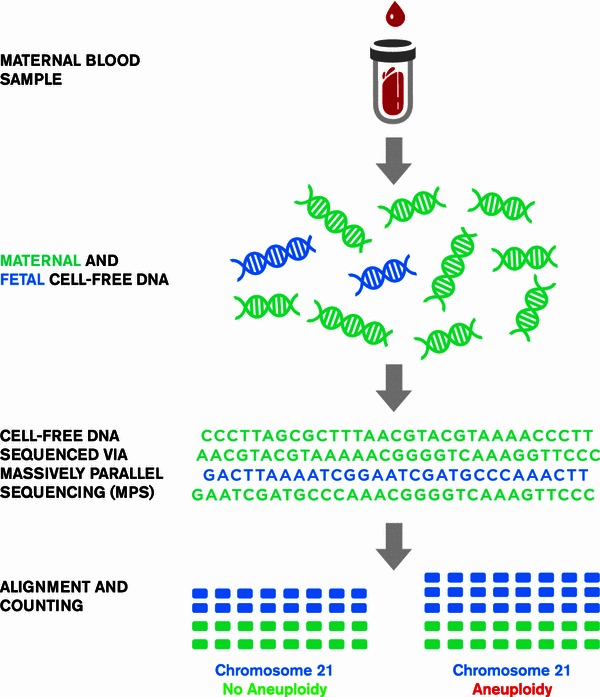
Massively parallel sequencing and counting for the detection of fetal aneuploidy. cfDNA is isolated from maternal plasma. The total cfDNA is sequenced by MPS, generating millions of sequence reads. Sequence reads are then aligned to sites from a reference human genome and the aligned reads (tags) are counted for determination of the chromosome ploidy status

In 2008, Fan et al. [14•] published a revolutionary paper describing a quantification method for non-invasive fetal aneuploidy detection involving counting chromosomes by mapping sequence tags generated via MPS of cfDNA in maternal plasma, even without separating maternal and fetal cfDNA or enriching for fetal cfDNA. More specifically, this method generates tens of millions of sequence reads across the entire genome that can be aligned and uniquely mapped (tagged) to sites from a reference human genome to identify their chromosome of origin. Once mapped, the tags can be counted for determination of the chromosome ploidy status (see Fig. 1). The ability to count millions of tags allows for very high sensitivity to detect aneuploidy in a given sample. When aneuploidy is present, there is an increase (trisomy) or decrease (monosomy) in the relative number of tags on the affected chromosome compared to the euploid chromosomes.

Ehrich et al. [27] and Chiu et al. [28] published additional studies on the performance of the above method for the detection of trisomy 21. As noted in their papers, the counting and normalization algorithms used in these studies using a z-score were unable to effectively detect other aneuploidies. This is likely due to technical and sample-to-sample sequencing variations in the chromosomal distribution of sequence reads as has been discussed in many papers [14•, 29•, 30]. Sehnert et al. [31•] and Bianchi et al. [32] developed and tested an optimized counting algorithm and demonstrated its ability to detect multiple chromosome abnormalities (e.g., trisomies 21, 18, 13, monosomy X, trisomy 20, trisomy 16) in two independent studies. Their optimized algorithm utilizes normalized chromosome values (NCVs), where the count of mapped tags for a given chromosome of interest is normalized to cumulative counts observed on a predetermined set of optimal (“reference”) chromosomes. This approach helps to correct for technical and sample-to-sample sequencing variations and mitigates the need to perform additional corrections on the data (e.g., correction for guanine–cytosine (GC) content) [7, 31•]. Subsequent studies by several groups have shown that trisomies 18 and 13 can also be detected by applying a GC adjustment to the *z*-score algorithm to help correct for variations in sequencing reads [29•, 30, 33].

Utilizing concepts that are similar to those developed by Fan et al. [14•], a second MPS counting approach sequencing only a limited number of cfDNA fragments has also been tested for non-invasive aneuploidy detection. This “targeted sequencing” approach has been demonstrated for chromosomes 21, 18 and 13 only [34–37].

## Clinical Validation Studies of NIPT

Three large-scale clinical validation studies originating in North America were performed from 2009 to 2011, and the results from these studies were reported in four recent publications [32, 33, 36, 38]. The following is an analysis of the study aspects we found to be of particular relevance to practicing clinicians: study design (sample and data collection, analysis plans) and measures of test performance (including test failures), as these directly correlate to clinical implementation of NIPT.

### Sample and Data Collection

All studies had multicenter participation and involved collection of blood from pregnant women who were undergoing invasive prenatal procedures (CVS or amniocentesis). Blood was drawn prior to performance of the procedures in all cases. Conventional cytogenetic karyotype from CVS or amniocentesis was used as the reference standard in all studies. Fluorescence in situ hybridization (FISH) or quantitative fluorescent polymerase chain reaction (QF-PCR) results from CVS/amniocentesis or on the products of conception were used as the reference standard in the few cases where a conventional metaphase karyotype was not available. To be eligible for the studies by Palomaki et al. [38] and Bianchi et al. [32], women were included if they were “high-risk” as defined by advanced maternal age, having a positive prenatal serum screen result, presence of an ultrasound finding suggestive of fetal aneuploidy and/or a history of aneuploidy. Norton et al. [36] accepted samples from any pregnant woman undergoing an invasive procedure. However, the sample size determinations, incidence of aneuploidy and participant demographics for that study indicate that the majority of their participants were also “high-risk”.

### Analysis Plans

Palomaki et al. [33, 38] and Bianchi et al. [32] used a blinded nested case–control approach while Norton et al. [36] was a cohort study that also used case–control analysis for determining test performance. All samples analyzed were from women with singleton pregnancies.

In the Palomaki et al. and Norton et al. studies, cases were the affected trisomy of interest (e.g., trisomy 21) and the controls were euploid samples only. Palomaki et al. [33, 38] did this first by analyzing the performance for trisomy 21 and subsequently analyzing trisomy 18 and trisomy 13 performance in a case–control analysis from the same initial cohort (along with reassessing the performance for trisomy 21 using a modified bioinformatics algorithm). Translocation trisomies and mosaics were not included in Palomaki et al., study. Norton et al., did independent case–control analyses for trisomies 21 and 18. They did not exclude cases of translocation trisomy but did exclude cases of mosaicism [36].

Bianchi et al., is the only study to simultaneously assess the performance of their NIPT for six independent categories in each eligible sample: trisomy 21, trisomy 18, trisomy 13, monosomy X and sex determination (XX or XY). In addition, since they included all samples with any abnormal karyotype and analyzed all chromosomes across the genome in the analyzed dataset, they also reported NIPT results for a variety of other chromosome abnormalities (e.g., trisomy 20) [32]. As a result of their study design, cases of translocation trisomy were included; cases with mosaicism were also sequenced but these were not included in the performance calculations. Controls for each analysis in this study were all of the analyzed samples that did not have the aneuploidy of interest. As such, the same sample with trisomy 21 could be counted as a “case” in one analysis and a “control” in another analysis.

Sample size requirements were determined prior to initiation of the studies. All of the studies were powered to include the number of trisomy 21 cases needed to achieve statistical significance at a high level of performance (sensitivity and specificity), approaching that of diagnostic tests such as CVS and amniocentesis which have sensitivities of 99.25 and 99.4 % and specificities of 98.65 and 99.5 %, respectively [3, 39]. Assumptions were based upon early, proof-of-principle studies anticipating that NIPT performance would meet or exceed prenatal screening tests as their goal.

### Test Performance

Trisomy and absence of trisomy were categorized as “consistent with trisomy” and “normal” in the Palomaki et al., studies, “affected” and “unaffected” in the Bianchi et al., study, and “high-risk” (risk score of ≥1 %) and “low-risk” (risk score <1 %) in the Norton et al., study [32, 33, 36, 38]. The sensitivities and specificities of NIPT for trisomy 21 and trisomy 18 are shown in Table 1. The sensitivities and specificities of NIPT for trisomy 13 are also shown in Table 1 for the Bianchi et al., and Palomaki et al., studies.

**Table 1:** Patient demographics and NIPT performance statistics

	Palomaki et al. [38]	Palomaki et al. [33]	Bianchi et al. [32]	Norton et al. [36]
Subjects enrolled (*N*)	4,664	4,664	2,882	4,002
Analyzed samples (*N*)	1,696	1,988*	532	3,007
Trisomy 21 cases (*N*)	212	212	89	81
Trisomy 18 cases (*N*)		59	36	38
Trisomy 13 cases (*N*)		12	14	
Monosomy X Cases (N)			16	
Female Cases (N)			233	
Male Cases (N)			184	
Maternal age cases and controls (years) mean ± SD	37 ± 5	36.6 ± 4.9 T18	34.4 ± 6.73	35.4 ± 7.3 T21
	36.6 ± 5.1	33.3 ± 5.6 T13	35.2 ± 6.40	34.5 ± 6.1 T18
		37.6 ± 5 eupld		34.3 ± 6.3 eupld
Gestational age mean cases and controls (weeks)	15.3	14.8 T18	14.8	16.4 T21
	15.0	15.2 T13	15.1	16.2 T18
		14.7 eupld		17.0 eupld
Trisomy 21 sensitivity (%)	98.6 (95.9–99.7)	99.1 (96.6–99.9)	100 (95.9–100)	100 (95.5–100)
Trisomy 21 specificity (%)	99.8 (99.4–99.9)	99.9 (99.7–99.9)	100 (99.1–100)	99.97 (99.8–99.99)
Trisomy 18 sensitivity (%)		100 (93.9–100)	97.2 (85.5–99.9)	97.4 (86.5–99.9)
Trisomy 18 specificity (%)		99.7 (99.3–99.9)	100 (99.2–100)	99.93 (99.75–99.98)
Trisomy 13 sensitivity (%)		91.7 (61–99)	78.6 (49.2–99.9)	
Trisomy 13 specificity (%)		99.1 (98.5–99.5)	100 (99.2–100)	
Female sensitivity (%)			99 (97.6–99.9)	
Female specificity (%)			99.5 (97.2–99.9)	
Male sensitivity (%)			100 (98–100)	
Male specificity (%)			100 (98.5–100)	
MX sensitivity (%)			93.8 (69.8–99.8)	
MX specificity (%)			99.8 (98.7–99.9)	

Numbers in parentheses are 95% confidence intervals given as %, *SD* standard deviation, *CI* confidence interval, *eupld* euploid, *MX* monosomy X

* 1695 of these samples were re-tested from original study [38]

It should be noted that Ashoor et al., also recently developed and optimized an algorithm for non-invasive trisomy 13 detection. The study design was significantly different than the aforementioned studies. Therefore, the data are not included in Table 1. This was a case–control study of ten cases of trisomy 13 (confirmed by CVS/amniocentesis) and 1,992 presumed euploid controls from a single site in the United Kingdom. They reported a calculated sensitivity of 80 % (95 % CI 48–94.9 %) and a specificity of 99.95 % (95 % CI 99.7–100 %) [37].

Results were not generated in any of the studies if the measured fetal fraction did not meet a certain threshold or if the assay failed at any step of the process. Test failure rates were reported by Palomaki et al. [33], Bianchi et al. [32] and Norton et al. [36] as 5.3, 3, and 4.6 %, respectively. Palomaki et al., was the only study to reflex to a second sample if the initial sample failed. They were able to reduce the number of test failures to 0.9 % if a second sample was tested [33]. It should be noted that Bianchi et al., categorized certain samples as “unclassified”. These samples were not included in the test failure rates as the assay provided a result, albeit an intermediate one [32].

All of the studies achieved high performance as seen by the sensitivity and specificity calculations (see Table 1). Based on the number of samples tested, the highest degree of confidence is seen for trisomy 21, followed by trisomy 18. Performance for chromosome 13 is impacted by smaller sample sizes and thus wider 95 % confidence intervals making it more difficult to draw conclusions.

## Commercially Available Tests in the United States

The highly successful results from the aforementioned clinical validation studies led to the recent launch of three commercially available non-invasive prenatal tests in the United States. Table 2 highlights the test similarities and differences in terms of which chromosomes are tested, sample acceptability criteria, and the timeframe in which results are returned.

**Table 2:** Comparison of commercially-available NIPTs

	Sequenom CMM^®^	Verinata Health^®^, an Illumina company	Ariosa™ Diagnostics
Chromosomes tested	21, 18, 13, XX, XY, MX, XXY, XXX, XYY	21, 18, 13 XXX, XXY, XYY, MX, X & Y are optional	21, 18, 13
Results reported as	Positive	Aneuploidy detected	High-risk (≥1 %)^*^
	Negative	No aneuploidy detected	Low-risk (<1 %)^*^
		Aneuploidy suspected/borderline value	
Gestational age at which test can be performed (weeks)	10+	10+	10+
Samples accepted for multi-fetal gestations	Yes	No	No
Samples accepted for egg donor/surrogate pregnancies	Yes	Yes	No
Turn-around time	8–10 days	8–10 days	8–10 days

* Risk score included

*MX* monosomy X

These tests are rapidly evolving and additional laboratories are predicted to offer non-invasive prenatal tests in the near future. As such, women’s healthcare providers are encouraged to contact the various laboratories to confirm the details of the tests prior to ordering. Several insurance plans have published coverage decisions that are now effective for all commercially-available NIPTs. Once individual laboratories offering NIPT sign test-specific contracts with insurance plans, their particular test will then be in-network and covered. This will mean lower out-of-pocket costs for many patients.

## Clinical Implementation

Providers can look to several professional societies for guidance on how to implement the new non-invasive tests for fetal aneuploidy into their practices. The International Society for Prenatal Diagnosis (ISPD), the National Society of Genetic Counselors (NSGC), the American College of Obstetricians and Gynecologists (ACOG) and the Society for Maternal Fetal Medicine (SMFM) have all commented on the use of NIPT in clinical practice [9••, 40, 41••]. In reviewing the various societal statements on NIPT, we identified three important themes that we feel are worthy of discussion.

1. There are several clinical indications for which NIPT should be considered.

After reviewing the published data on NIPT, the aforementioned professional societies have unanimously agreed that NIPT is a safe and effective screening test for fetal aneuploidy in high-risk women. It can be used as a primary screen for women at high-risk based on their age, the presence of ultrasound anomalies, a history of aneuploidy and in those pregnancies at risk for aneuploidy due to the presence of a Robertsonian translocation in a parent. NIPT can also be used as a follow-up test for those women who have a positive first and/or second trimester screen.

None of the societies support the use of non-invasive prenatal tests in the low/average risk populations at this time due to the lack of data. Furthermore, ACOG does not support its use in multi-fetal gestations.

2. NIPT is an advanced screening tool and confirmation of positives through CVS/amniocentesis is currently necessary.

Given the high sensitivity of NIPT, patients with negative NIPT results should be counseled that the chance of aneuploidy for the chromosomes tested is low. However, a negative result does not completely rule out the possibility of trisomies 21, 18 and 13 and NIPT results should be used in the context of all relevant clinical information. Furthermore, NIPT does not currently test for all aneuploidies, nor does it provide information on polyploidy and single-gene disorders. As such, patients should continue to be given the option of diagnostic testing (CVS or amniocentesis), especially in cases of an ultrasound-identified fetal structural abnormality or a family history of a genetic condition.

Positive NIPT results are typically associated with an affected fetus. However, there will be instances where a patient has a positive NIPT result and a normal CVS/amniocentesis result. Therefore, patients receiving a positive result should be counseled about the importance of confirming the result via CVS or amniocentesis, especially prior to making irreversible pregnancy management decisions.

Clinicians will encounter situations where patients have a positive NIPT result and decline confirmatory testing. For patients who decline invasive testing and continue the pregnancy, confirmation of a positive NIPT result can be done postnatally. If after counseling, a patient chooses to decline confirmatory CVS/amniocentesis and proceeds with termination of the pregnancy, chromosome analysis should be performed on the products of conception. Not only will this allow for confirmation of the NIPT result, it will assist with determination of recurrence risks as NIPT does not distinguish non-disjunction trisomy from translocation trisomy.

When a positive result from NIPT is inconsistent with a CVS or amniocentesis karyotype, several biological explanations for such discordant results should be considered. For example, as it has been hypothesized that fetal cfDNA originates from placental cytotrophoblasts [42, 43], it is possible that the NIPT result detected confined placental mosaicism (CPM). Also, given that NIPT analyzes total cfDNA (maternal and fetal), a positive NIPT finding may in fact be detecting maternal aneuploidy, full or mosaic. Other biological explanations include low-level fetal mosaicism that is undetectable by routine cytogenetics and the presence of fetal cell-free DNA from a demised/vanishing co-twin [7]. Additionally, there will be a very small percentage of positive NIPT results that are falsely positive due to the technology itself.

3. It is crucial that patients make informed choices about undergoing NIPT.

Informed choice is a process that stems from the bioethical principle of respect for autonomy. Informed choice implies that patients are given adequate information about the risks and benefits of a procedure and are free to make choices about the procedure based on the information provided and their own personal values and beliefs [44, 45••]. It is common practice that high-risk pregnant women who are considering amniocentesis or CVS undergo an informed choice process with a genetic counselor as these procedures are associated with maternal and fetal risks and provide definitive information about certain medical conditions. It is also common practice that pregnant women considering aneuploidy screening tests simply receive pretest information (usually minimal) from their primary provider, and only meet with a genetic counselor if their screening result is positive [45••]. Since NIPT does not pose a physical risk to the mother or her fetus, some healthcare providers question whether patients need to make informed choices prior to undergoing such testing [46].

Even though NIPT is not risky like CVS and amniocentesis, the implications of a positive NIPT result are significant. Given the high specificity of NIPT, most women who receive a positive result will have essentially received a prenatal diagnosis of aneuploidy. This is much different than conventional screening tests which have a significant false positive rate, affording women time to decide whether they truly want to know certain information about their fetus’s health prenatally. Therefore, giving patients minimal information prior to such tests, as is done with current aneuploidy screens, would according to Benn and Chapman, “[B]e a much more seriously deficient medical practice, undermining patient autonomy and reproductive decision-making” [45••].

## Future Directions

In the past 5–10 years, we have seen the clinical introduction of NIPT for RhD status, the determination of fetal sex to aid in the assessment for sex-linked conditions, and certain fetal aneuploidies. The utility of MPS of cfDNA is currently being evaluated for multi-fetal gestations as well as for average-risk pregnant women. This is incredibly exciting, but what is perhaps most exciting is the very real possibility that the MPS of cfDNA technique will be clinically available and effective for all fetal aneuploidies, sub-chromosomal deletions and duplications, monogenic disorders and eventually the entire fetal genome. Furthermore, there is promising research in the field of using quantitative cfDNA testing as a biomarker to provide early diagnosis of preeclampsia and other pregnancy complications [47, 48]. This suggests that analysis of cfDNA may be able to predict maternal, as well as fetal, well-being during pregnancy.

## Conclusion

Cumulative evidence suggests that NIPT using MPS can be safely introduced into existing prenatal screening algorithms to reduce unnecessary invasive procedures. Guidelines from several professional societies now exist to aid women’s healthcare providers in determining under which circumstances patients should be offered such testing. In turn, it is now the responsibility of the providers to differentiate between the clinically available non-invasive prenatal tests, select the most efficacious NIPT for their patient population and ensure that pre- and post-test genetic counseling is effectively provided.
